# Probable DRESS syndrome induced by IL-1 inhibitors

**DOI:** 10.1186/s13023-017-0645-x

**Published:** 2017-05-11

**Authors:** L. Polivka, J. S. Diana, A. Soria, C. Bodemer, P. Quartier, S. Fraitag, B. Bader-Meunier

**Affiliations:** 1 Paris Descartes-Sorbonne Paris Cité University and IMAGINE Institute, Paris, France; 2 Department of Pediatric Hematology-Immunology and Rheumatology, Necker-Enfants Malades Hospital (AP-HP), 149 rue de Sèvres, 75015 Paris, France; 3 Sorbonne University, UPMC University Paris 06, Immunology and Infectious diseases Center – Paris (Cimi-Paris), INSERM U1135, Paris, France; 4 Department of Dermatology and Allergology, Tenon Hospital (AP-HP), Paris, France; 5 Department of Dermatology, Necker-Enfants Malades Hospital (AP-HP), Paris, France; 6 Department of Pathology, Necker-Enfants Malades Hospital (AP-HP), Paris, France

**Keywords:** DRESS, IL-1 inhibitors, Autoinflammatory disease

## Abstract

Interleukin (IL)-1 inhibitors have been increasingly used for treating autoinflammatory diseases during the last 10 years, but the spectrum of their possible side effects is not yet fully known. Here, we bring physicians’ attention to a new severe complication of IL-1 inhibitors, manifesting as a probable drug reaction with eosinophilia and systemic symptoms (DRESS) in two patients.

**Dear Editor,**

We would like to comment the paper of L. Rossi-Semerani et al. published in *Orphanet Journal of Rare Diseases* and to bring readers’ attention to a new severe complication of interleukin (IL)-1 inhibitors [1]. In this recent cross-sectional survey of the off label use of IL-1 inhibitors for treatment of auto-inflammatory diseases in 189 patients, the authors reported that anti-IL1 agents were well tolerated in most patients. The main adverse events being mild injection-site reaction. This good safety profile of IL-1-inhibitors was confirmed by Vitale et al. [2].

However, for the first time to our knowledge, we report on two cases of probable drug reaction with eosinophilia and systemic symptoms (DRESS) syndrome in patients treated with IL-1 inhibitors for a systemic autoinflammatory condition of undetermined cause.

Patient #1 was a 2-year-old girl born to non-consanguineous parents. Since the age of 12 months, she had presented with recurring episodes of unexplained fever, urticaria (Fig. 1a), arthralgia, poor general health status, leukocytosis and elevated serum C-reactive protein (CRP). There was no evidence of infection and these features were consistent with the diagnosis of autoinflammatory disease (AID). Mutations in *MVK* and *NLRP3* genes were excluded. After the failure of treatment with nonsteroidal anti-inflammatory drugs and anakinra, subcutaneous canakinumab (4 mg/kg monthly) was effective for the first two months of treatment. Ten days after the third injection of canakinumab (half-life: 24 days), Patient #1 developed widespread exanthema, pruritus (Fig. 1b), fever, severe eosinophilia (10000/mm^3^), elevated serum CRP, and slightly elevated serum liver enzyme levels. There was no lymphadenopathy, or other organ involvement. A skin biopsy revealed confluent keratinocyte necrosis and a moderate perivascular lymphocytic infiltrate (Fig. 1d and e). According to PCR assays, she was positive for human herpesvirus 6 (HHV6, 1000 copies/ml) and negative for Epstein–Barr virus (EBV) and cytomegalovirus (CMV). The DRESS score (RegiSCAR) was 5 out of 9, corresponding to “probable” DRESS syndrome [3]. Accordingly, treatment with intravenous methylprednisolone (2 mg/kg/day) was initiated, and canakinumab was withdrawn. This resulted in a complete resolution of symptoms within 14 days. This remission persisted while oral prednisolone was slowy tapered.

**Fig. 1:**
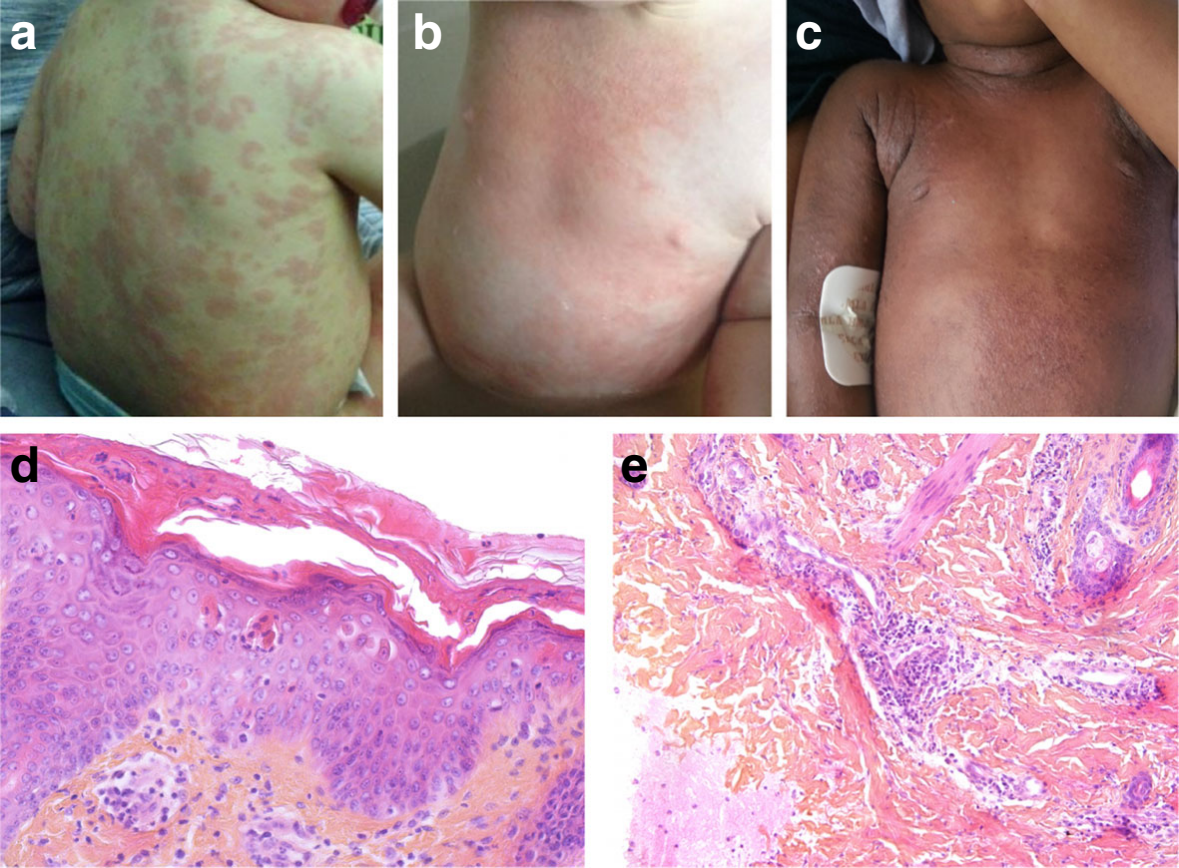
Clinical and histopathological findings of patients #1 and #2. **a** Patient #1: urticaria during flares. **b** Patient #1: widespread exanthema after three injections of canakinumab. **c** Patient #2: skin rash, seven days after the initiation of anakinra. **d**, **e** Patient #1: histologic assessment of the skin biopsy, showing confluent keratinocyte necrosis (**d**) and moderate perivascular lymphocytic infiltrate (**e**)

Patient #2 was a two-year-old girl. Since the age of 15 months, she had presented with recurring episodes of fever and urticaria. At the age of 16 months, she developed macrophage activation syndrome (MAS) associated with primary EBV infection. MAS resolved within one month, following treatment with two doses of etoposide, cyclosporine and corticosteroids. One month later, she developed new flares of urticaria, fever and elevated serum levels of inflammatory markers. There was no evidence of infection, nor mutations in *MVK*, *NLRP3* and *NLRC4* genes. The normal expression of perforin in cytotoxic granules and the normality of degranulation test excluded most of the causes of familial hemophagocytic lymphohistiocytosis. Combination treatment with anakinra (2 mg/kg/day) and corticosteroids (1 mg/kg/day) was effective within one day. Seven days after the initiation of anakinra (half-life: 4 to 6 h), Patient #2 presented with widespread exanthema (predominantly effecting the skin folds) (Fig. 1c), fever, asthenia, lymphadenopathy and eosinophilia (5000/mm^3^). She was PCR-positive for EBV (2000 copies/ml) and CMV (500 copies/ml). A skin biopsy revealed a mild keratinocyte necrosis and a dermal eosinophilic infiltrate. The DRESS (RegiSCAR) score was 5 corresponding to “probable” DRESS syndrome. Anakinra was withdrawn, and topical corticosteroids were initiated and were effective within 7 days.

DRESS syndrome is a rare, life-threatening, adverse drug reaction associated primarily with the administration of anticonvulsants, allopurinol and antibiotics [4]. Given the mortality rate of up to 10% associated with DRESS, it is essential that physicians recognize this condition. The main symptoms (skin rash, fever, hematologic abnormalities (such as eosinophilia and atypical lymphocytes), and internal organ involvement) usually appear within 1 week to 8 weeks of exposure to the culprit drug. Given the heterogeneity of the skin eruptions and the variety of organs involved, the diagnosis of DRESS is challenging. Accordingly, Kardaun et al. have developed an accountability score for DRESS, which ranged from −4 to 9 (score <2: no DRESS, score 2–3: possible DRESS, score 4–5: probable case, score >5: definite DRESS) [3]. Thus, this score allowed to classify this severe adverse drug reaction (ADR) as a probable DRESS syndrome in the two patients. Although the histological lesions of DRESS syndrome are not specific, the keratinocytes damage and the dermal inflammatory infiltrate in the patient’s biopsies were compatible with this diagnosis [5].

Although the exact pathophysiologic mechanism of DRESS is not fully understood, two key elements are thought to be involved: (i) the reactivation of herpes virus family members (especially EBV, CMV, HHV7 and HHV6), and (ii) genetic predisposition in people with certain HLA alleles. In particular, associations have been shown for allopurinol (HLA-B*58:01)- and carbamazepine (HLA-A*3101)-induced DRESS syndrome. However, we did not ascertain the HLA type for each patient.

According to the similarity of the autoinflammatory manifestations in our two patients, we cannot exclude that they shared the same genetic disease which might predispose to severe ADR after anti-IL1 exposure.

We emphasized that DRESS syndrome may occur after treatment with anti-IL1 for unclassified AID. Further genetic investigations are required in our patients to determine if a genetic susceptibility to severe ADR is involved in this severe complication.

## Abbreviations

ADR: Adverse drug reaction
AID: Autoinflammatory disease
CMV: Cytomegalovirus
CRP: C-reactive protein
DRESS: Drug reaction with eosinophilia and systemic symptoms
EBV: Epstein–Barr virus
HHV6: Human herpesvirus 6
HHV7: Human herpesvirus 7
IL: Interleukin
MAS: Macrophage activation syndrome
